# Effects and mechanisms of basic fibroblast growth factor on the proliferation and regenerative profiles of cryopreserved dental pulp stem cells

**DOI:** 10.1111/cpr.12969

**Published:** 2020-12-17

**Authors:** Lihua Luo, Yanni Zhang, Hongyu Chen, Fengting Hu, Xiaoyan Wang, Zhenjie Xing, Abdullkhaleg Ali Albashari, Jian Xiao, Yan He, Qingsong Ye

**Affiliations:** ^1^ School and Hospital of Stomatology Wenzhou Medical University Wenzhou China; ^2^ Department of Stomatology Ningbo Women and Children Hospital Ningbo China; ^3^ School of Pharmaceutical Sciences Wenzhou Medical University Wenzhou China; ^4^ Laboratory of Regenerative Medicine Tianyou Hospital Wuhan University of Science and Technology Wuhan China; ^5^ Center of Regenerative Medicine Renmin Hospital of Wuhan University Wuhan China

**Keywords:** basic fibroblast growth factor, cell culture technique, cryopreservation, dental pulp stem cells, extracellular signal‐regulated kinase pathway, transient receptor potential canonical 1 channel

## Abstract

**Objectives:**

Various factors could interfere the biological performance of DPSCs during post‐thawed process. Yet, little has been known about optimization of the recovery medium for DPSCs. Thus, our study aimed to explore the effects of adding recombinant bFGF on DPSCs after 3‐month cryopreservation as well as the underlying mechanisms.

**Materials and methods:**

DPSCs were extracted from impacted third molars and purified by MACS. The properties of CD146^+^ DPSCs (P3) were identified by CCK‐8 and flow cytometry. After cryopreservation for 3 months, recovered DPSCs (P4) were immediately supplied with a series of bFGF and analysed cellular proliferation by CCK‐8. Then, the optimal dosage of bFGF was determined to further identify apoptosis and TRPC1 channel through Western blot. The succeeding passage (P5) from bFGF pre‐treated DPSCs was cultivated in bFGF‐free culture medium, cellular proliferation and stemness were verified, and pluripotency was analysed by neurogenic, osteogenic and adipogenic differentiation.

**Results:**

It is found that adding 20 ng/mL bFGF in culture medium could significantly promote the proliferation of freshly thawed DPSCs (P4) through suppressing apoptosis, activating ERK pathway and up‐regulating TRPC1. Such proliferative superiority could be inherited to the succeeding passage (P5) from bFGF pre‐stimulated DPSCs, meanwhile, stemness and pluripotency have not been compromised.

**Conclusions:**

This study illustrated a safe and feasible cell culture technique to rapidly amplify post‐thawed DPSCs with robust regenerative potency, which brightening the future of stem cells banking and tissue engineering.

## Graphical abstract

The graphical abstract demonstrated that short‐term application of bFGF in the early stage post‐thawing could rescue cellular viability without shifting their pluripotency, unexpectedly, this proliferative superiority could prolong to the succeeding passage, so the usage time of bFGF for post‐thawed cell culture should be addressed. This study illustrated a safe and feasible cell culture technique to rapidly amplify post‐thawed DPSCs with robust regenerative potency, which brightening the future of stem cells banking and tissue engineering.

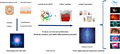

## 1 INTRODUCTION

Dental pulp stem cells (DPSCs), derived from ectomesenchyme of the neural crest, are firstly isolated from permanent tooth in 2000.^1^, ^2^ As a type of mesenchymal stem cell (MSCs), DPSCs have the ability to regenerate dental pulp, skin, cartilage, fat and so on.^3^ Notably, up‐regulated expression of CD146 in MSCs could enhance cellular proliferation and trilineage differentiation.^4^ Therefore, CD146 has been recognized as a biomarker for MSCs from adult and foetal organs. Otherwise, to satisfy the growing requirements from basic and clinical research, dental stem cells bank has emerged to store DPSCs.^5^ Cryopreservation is a standard way to bank cells. However, cryopreservation can cause problems. For instance, apoptosis is often observed in post‐thawed stem cells induced by the activation of caspase pathway,^6^ and cells suffer from poor cellular viability, proliferation, anti‐oxidation and pluripotency, which are probably resulted by altering the spatial configuration of cell membrane proteins during the cryopreservation.^6^, ^7^, ^8^ Nevertheless, it is critical to maintain the core properties of DPSCs, including proliferation, stemness and pluripotency. And during this delicate recovery period, a slight alteration in the medium formulation may lead to a distinctly different cellular fate. Yet, few studies have focused on optimizing the recovery medium for DPSCs.

Some studies have shown that many growth factors including the human recombinant growth factors (eg, basic fibroblast growth factor, bFGF)^9^, ^10^ and the autogenous growth factors (eg, platelet‐rich plasma, PRP or platelet lysate, PL)^10^, ^11^, ^12^ have ability to affect the biology properties of MSCs. Among them, PRP or PL is a blood‐derived concentrate with a combination of growth factors, and has been confirmed to promote the proliferation and differentiation of stem cells.^11^, ^12^, ^13^ However, the products of PRP or PL display a number of disadvantages, and for instance, the preparation procedure is very complex and expensive, the composition may vary according to the different patients and preparation methods, and the mechanism and mainly responsible factors’ effect on stem cells have not been clear.^10^, ^13^ Recombinant human basic fibroblast growth factor (bFGF) is a sort of polypeptides with the capability to enhance cellular proliferation and life span.^14^ When DPSCs are applied with bFGF, their neural differentiation potential is enhanced and a synergistic role could display in the repair of central nervous system.^15^, ^16^ Short‐term bFGF treatment also could enhance stemness of dental stem cells by regulating the expression of STRO‐1 and intrinsic markers such as Nanog, Oct4, Sox2 and Rex1.^17^, ^18^ Moreover, bFGF functions through its specific receptors (FGFRs), activated FGFRs transduce the signals by two dominant pathways including RAS‐mitogen‐activated protein kinase (MAPK) pathway and phosphatidylinositol‐4,5‐bisphosphate 3‐kinase/AKT (PI3K/AKT) pathway.^19^ Exhibition of the pluripotency requires MAPK/extracellular signal‐regulated kinase (ERK) signalling, whereas PI3K/AKT signals boost when stem cells become lineage‐restricted.^20^ In addition, bFGF prevents apoptosis partially depending on the ERK pathway to regulate Bcl‐2/Bax/Caspase‐3 signals.^21^ Therefore, bFGF plays a crucial role in the vitality, stemness and pluripotency of stem cells.

Besides, ERK pathway regulates cell proliferation.^22^ Transient receptor potential canonical 1 (TRPC1) channel takes part in the proliferation of MSCs, endothelial/neural progenitor cells and cochlear spiral ganglion stem cells.^23^, ^24^, ^25^, ^26^ And TRPC1 channel also mediates bFGF/FGFR‐1‐induced Ca2^+^ entry during the neural stem cells proliferation.^27^ Activated TRPC1 channel causes Ca2^+^ entry, leading to the trigger of ERK/cAMP response element‐binding protein (CREB) pathway and enhanced cell proliferation.^28^ Nonetheless, it is less known about how bFGF affects the proliferation of post‐thawed DPSCs.

Hence, in this study, we first explored the minimal effective dose of bFGF on post‐thawed DPSCs, with which it could promote cellular viability within a short time, yet not compromise regenerative property of the following passages. In addition, we unveiled the relation between TRPC1 channel and ERK pathway in cellular proliferation of post‐thawed DPSCs with the presence of bFGF.

## 2 MATERIALS AND METHODS

### 2.1 Isolation and culture of DPSCs (1 month)

The procedure was described previously.^29^ Briefly, impacted third molars were obtained from 18 to 30 years old patients at the Department of Oral and Maxillofacial Surgery, Stomatological Hospital of Wenzhou Medical University. Dental pulps from five donors were pooled for DPSCs, marked as primary culture (P0). First, pulp tissue was separated from tooth and digested with 3 mg/mL collagenase type I (Invitrogen‐Gibco, Carlsbad, USA) and 4 mg/mL dispase (Sigma‐Aldrich, Darmstadt, Germany) for 30 minutes at 37°C. Then, this cellular suspension and pulp tissue were cultivated in T25 flasks with α‐Modified Eagle's Medium (α‐MEM) containing 20% foetal bovine serum (FBS), 100 U/mL penicillin and 100 μg/mL streptomycin (Gibco, Carlsbad, USA) at 37°C in 5% CO_2_ incubator. The medium was replaced on day 5 followed by a routine medium change every 3 days. Upon 85% confluency, this heterogeneous DPSCs population was detached using 0.25% trypsin/ethylene diamine tetraaceticc acid (EDTA, Gibco, Carlsbad, USA). Complete culture medium, α‐MEM supplemented with 10% FBS, 100 U/mL penicillin and 100 μg/mL streptomycin were used to culture DPSCs starting from passage 1 (P1) and changed every 3 days. The work has been approved by the Ethics Committee of Wenzhou Medical University (Project No. 2018008). Workflow and study timeline were shown in Figure S1.

### 2.2 Purification, identification and proliferation of CD146^+^ DPSCs (1 week)

In order to obtain CD146‐positive (CD146^+^) DPSCs, the heterogeneous cells were populated till P2 and purified by the magnetic‐activated cell sorting (MACS) method.^29^ Upon 90% confluence, DPSCs (P2) were harvested in T75 flasks, and 1 × 10^7^ cells were labelled and separated using CD146 MicroBead Kit (Miltenyi Biotec GmbH, Bielefeld, Germany) according to manufacturer's instructions.

After purification, CD146^+^ DPSCs were seeded in T75 flasks, marked as P3. Upon 90% confluence, 1 × 10^6^ cells were aliquoted into EP tubes and incubated on ice for 30 minutes in the dark with fluorescent‐conjugated mouse monoclonal antibodies [dilution 1:20] including the following: anti‐human CD73‐phycoerythrin (PE), anti‐human CD90‐PE/Cyanine7, anti‐human CD34‐fluorescein isothiocyanate (FITC), anti‐human CD14‐FITC and anti‐human leucocyte antigen D‐related (HLA‐DR)‐PE. IgG1‐PE, IgG1‐PE/Cyanine7, IgG1‐FITC, IgG2a‐FITC and IgG2a‐PE were used as isotype controls. All antibodies were purchased from BioLegend, San Diego, United States. These markers were detected by CytoFLEX Flow Cytometer (Beckman Coulter, California, USA). Data analysis was performed using CytExpert Software version 2.3.

The cell proliferation of CD146^+^ DPSCs was determined by the Cell Counting Kit‐8 (CCK‐8) assay. CD146^+^ DPSCs were cultivated in 96‐well plates from the passage 3 (P3) to the passage 7 (P7), respectively. At the designated culture time points, 10 µL/well of CCK‐8 solution (Dojindo Molecular Technologies, Kumamoto, Japan) was added and incubated for 1 hour at 37°C in 5% CO_2_ incubator. The optical density (OD) values were measured at 450 nm by an absorbance microplate reader (Varioskan LUX, ThermoFisher, USA). Meanwhile, the cell population doubling time of CD146^+^ DPSCs (P3 or P4) was calculated as the following formula ^30^: cell population doubling time = (*t* × log 2)/(log *N*2 − log *N*1), where *t* was the number of culture days, *N*1 was the number of cells at the beginning of culture phase, and *N*2 was the number of cells at the end of culture phase. In this study, *t* was designated as 1, 3, 5 and 7 days, respectively.

### 2.3 Cryopreservation and recovery of DPSCs (3 months)

The CD146^+^ DPSCs (P3) in residual T75 flasks were harvested and re‐suspended with freezing medium, consisting of 10% dimethyl sulphoxide (DMSO, Sigma‐Aldrich, Steinheim, Germany) and 90% FBS. 1 × 10^6^ cells were aliquoted into cryovials (Corning, Lowell, MA) and stored in the Nalgene^™^ Cryo 1°C Freezing Container (Thermo Fisher Scientific, USA), then kept in −80°C Freezer overnight for gradient cooling enabling a cooling rate at −1°C/min. Finally, they were transferred into liquid nitrogen.

In 3 months, CD146^+^ DPSCs were thawed in water bath at 37°C and seeded in 96‐well plates (2 × 10^3^ cells per well), marked as P4. Complete culture medium was used as the control medium (CM). 5 ng/mL bFGF was chosen as the minimal concentration in this experiment according to the literature, which showed highly proliferative response from bone marrow mesenchymal stem cells (BMMSCs).^31^ Moreover, a study had been reported that 100 ng/mL bFGF had obvious impact on the proliferation of dental pulp cells.^32^ Thus, in experiment groups, recovered DPSCs (P4) were immediately supplemented with 5, 10, 20, 50, 80 and 100 ng/mL bFGF. Fresh medium with bFGF was changed every 2 days.

### 2.4 Cellular proliferation of post‐thawed DPSCs (1 week)

DPSCs (P4) were cultivated in 96‐well plates for 1, 3, 5 and 7 days. The effect of bFGF on cellular viability was determined by the Cell Counting Kit‐8 (CCK‐8) assay.

### 2.5 Apoptosis, ERK pathway and TRPC1 channel of post‐thawed DPSCs (1.5 weeks)

Based on the previous cellular viability study, 20 ng/mL bFGF was chosen to investigate its influence on apoptosis, ERK pathway and TRPC1 channel by Western blotting as described previously.^15^ Briefly, freshly recovered DPSCs (P4) were seeded into 6‐well plates (6 × 10^3^ cells per well) and supplemented with 20 ng/mL bFGF for 3 days. Lysate proteins were extracted from cells via RIPA buffer (Beyotime, Shanghai, China) containing PMSF (protease inhibitor), then quantified by BCA Assay Kit (Beyotime, Shanghai, China). 20 μg protein was added to 10% SDS‐PAGE gel and transferred to a PVDF membrane. After blocking nonspecific binding sites, blots were incubated with following primary antibodies at 4°C overnight: anti‐Bcl‐2, anti‐phospho‐Erk1/2 (p‐Erk1/2), anti‐Erk (1:1000, Cell Signaling Technology, Inc, USA), anti‐Bax (1:2000, Abcam, Cambridge, UK) and anti‐TRPC1 (1:300, Santa Cruz Biotechnology, Santa Cruz, USA). Then, Horseradish peroxidase–conjugated secondary antibody (1:10 000, Biosharp, Anhui, China) was added and incubated for 1 hour at room temperature. Proteins were visualized with an Efficient Chemiluminescence (ECL) Kit (Biological Industries, Kibbutz Beit Haemek, Israel). Target proteins were detected by ChemiDoc XRS and Imaging System (Bio‐Rad Laboratories, Hercules, USA). The integrated density of each band was digitalized and measured via Image J Software version 1.8.0 (National Institutes of Health, Bethesda, USA). Therewith, the target band was normalized using GAPDH band as the housekeeping protein (n = 3 per group).

### 2.6 Cellular proliferation of post‐thawed DPSCs after TRPC1 inhibition (0.5 week)

Freshly recovered DPSCs (P4) were plated into 96‐well plates (2 × 10^3^ cells per well) and incubated with complete culture medium overnight at 37°C. Then, 0, 1, 5, 10, 20 and 25 μmol/L TRPC1 inhibitor (SKF‐96365, Sigma‐Aldrich, Steinheim, Germany) were added and incubated for 24 hours. Afterwards, fresh complete medium was replaced in the control group, and 20 ng/mL bFGF was supplemented in experiment groups. In 24 hours, cell viability was quantified by CCK‐8 assay.

### 2.7 Cellular proliferation of the succeeding passage from bFGF pre‐treated DPSCs (1 week)

Freshly recovered DPSCs (P4) were plated into 6‐well plates (6 × 10^3^ cells per well) and supplemented with 20 ng/mL bFGF for 5 days in experiment groups. Complete medium was used in control group. Upon 90% confluence, DPSCs were detached and seeded in 96‐well plates (2 × 10^3^ cells per well), marked as P5. Afterwards, these DPSCs (P5) were cultivated with bFGF‐free culture medium for 1, 3, 5 and 7 days, and cellular viability was quantified by CCK‐8 assay.

### 2.8 Stemness of the succeeding passage from bFGF pre‐treated DPSCs (2.5 weeks)

In experiment groups, freshly recovered DPSCs (P4) were supplemented with 20 ng/mL bFGF for 7 days. Then, cells were passaged and cultured with bFGF‐free culture medium (P5). Fresh medium was changed every 2 days. Upon 90% confluence, cellular stemness was analysed via immunofluorescent staining, flow cytometry and Western blotting.

As for immunofluorescent staining, DPSCs (P5) were seeded on cytoslides in 6‐well plates previously, fixed with 4% paraformaldehyde and blocked with 5% bovine serum albumin (BSA, Solarbio, Beijing, China) containing 0.1% Triton X‐100 (Solarbio, Beijing, China) for 30 minutes. Then, they were incubated with primary antibodies: rabbit anti‐CD146 (1:200, Abcam, Cambridge, UK) and mouse anti‐STRO‐1 (1:50, Santa Cruz Biotechnology, Santa Cruz, USA) at 4°C overnight. Afterwards, secondary antibodies: Alex Fluor 488 conjugated anti‐rabbit IgG and Alex Fluor 555 conjugated anti‐mouse IgG (Abcam, Cambridge, UK) were incubated for 1 hour in the dark. Nuclei were counterstained by DAPI (Beyotime, Shanghai, China). These cytoslides were detected by fluorescence microscopy (DMi8, Leica, Germany). The fluorescent intensity was analysed by Image‐Pro Plus Software version 6.0 (Media Cybernetics, Maryland, USA) and normalized with the control group (CM, bFGF‐free) (n = 3 per group).

As for flow cytometry, briefly, DPSCs (P5) were detached from T75 flasks and incubated with antibodies including the following: anti‐human CD73‐PE, anti‐human CD90‐PE/Cyanine7, anti‐human CD14‐FITC and HLA‐DR‐PE (BioLegend, San Diego, USA). Isotype antibodies were used as negative controls. Data were analysed by CytExpert Software version 2.3 (Beckman Coulter, California, USA).

As for Western blotting, briefly, DPSCs (P5) were cultured in 6‐well plates (6 × 10^3^ cells per well). Proteins were incubated with primary antibodies including the following: anti‐CD146 (1:10 000) and Nanog (1:1000) (Abcam, Cambridge, UK) at 4°C overnight. Secondary antibody (1:10 000, Biosharp, Anhui, China) was incubated for 1 hour at room temperature. Target proteins were tested by ChemiDoc XRS and Imaging System (Bio‐Rad Laboratories, Hercules, USA).

### 2.9 Pluripotency of the succeeding passage from bFGF pre‐treated DPSCs (1 month)

DPSCs were induced to neurons, osteoblasts and adipocytes using previous protocols.^15^, ^33^, ^34^ Briefly, DPSCs (P5) were seeded in 6‐well plates (6 × 10^3^ cells per well). In neurogenic differentiation, cells were cultured according to the chemical‐induced protocol without growth factors,^34^ contained serum‐free DMEM‐high glucose (DMEM‐HG) (Gibco, Carlsbad, USA) supplemented with 50 μg/mL ascorbic acid‐2‐phosphate, 10^−7^ mol/L dexamethasone, 50 μmol/L indomethacin, 10 μg/mL insulin and 0.45 mmol/L 3‐isobutyl‐1‐methyl‐xanthine (Thermo Fisher Scientific, Santa Clara, USA) for 6 days, and the medium was changed every 3 days. Then, the immunofluorescent staining was performed with Nestin (1:1000), GFAP (1:300), β‐tubulin III (1:3000) and NeuN (1:200) (Sigma‐Aldrich, Steinheim, Germany). In osteogenic and adipogenic differentiation, the OriCell™ MSCs osteogenic and adipogenic induction medium (Cyagen, Santa Clara, USA) were added to 90% confluent DPSCs (P5) according to the manufacturer's instructions. After 3 weeks, cells were stained with Alizarin Red and Oil Red O, respectively. Then, stained Oil Red O and stained Alizarin Red were taken photographs under the inverted microscope (TS100, Nikon, Japan), and quantified by dissolving in 100% isopropanol and 10% cetylpyridinium chloride buffer, respectively. Afterwards, the OD values of the solution were separately measured at 500 nm and 550 nm by a microplate reader (Varioskan LUX, ThermoFisher, USA).

### 2.10 Statistical analysis

All data were presented as mean ± standard deviation (SD). One‐way ANOVA was used for comparisons among groups. Tukey's test or Dunnett *post hoc* test and Student's *t* test were used for comparisons between groups. *P* < .05 was considered as statistically significant. Statistical analyses were performed using the spss 19.0 statistics software (SPSS, Chicago, IL).

## 3 RESULTS

### 3.1 The biological properties of DPSCs

The procedure from isolation to identification of DPSCs was demonstrated in Figure S2A. Cells (P0) penetrated out from pulp tissue pieces on day 7 (Figure S2B), and high density colonies were formed on day 10 (Figure S2C). After primary culture, this heterogeneous cell population was expanded till passage 2. Then, CD146^+^ DPSCs (P3) were sorted by MACS and characterized by flow cytometry (FC). They displayed the typical fibroblast‐like morphology (Figure S2D), and positively expressed CD73 and CD90 (<95%), lacking the expressions of CD34, CD14 and HLA‐DR (<2%) (Figure S2E). After cultured for 3 days, the cell proliferation of DPSCs had no significant difference from the passage 3 (P3) to the passage 7 (P7) (Figure S3). Furthermore, the doubling time of CD146^+^ DPSCs was calculated as 13‐31 hours based on our pilot experiments.

### 3.2 Post‐thawed DPSCs suffered from proliferation deceleration

**Figure 1:**
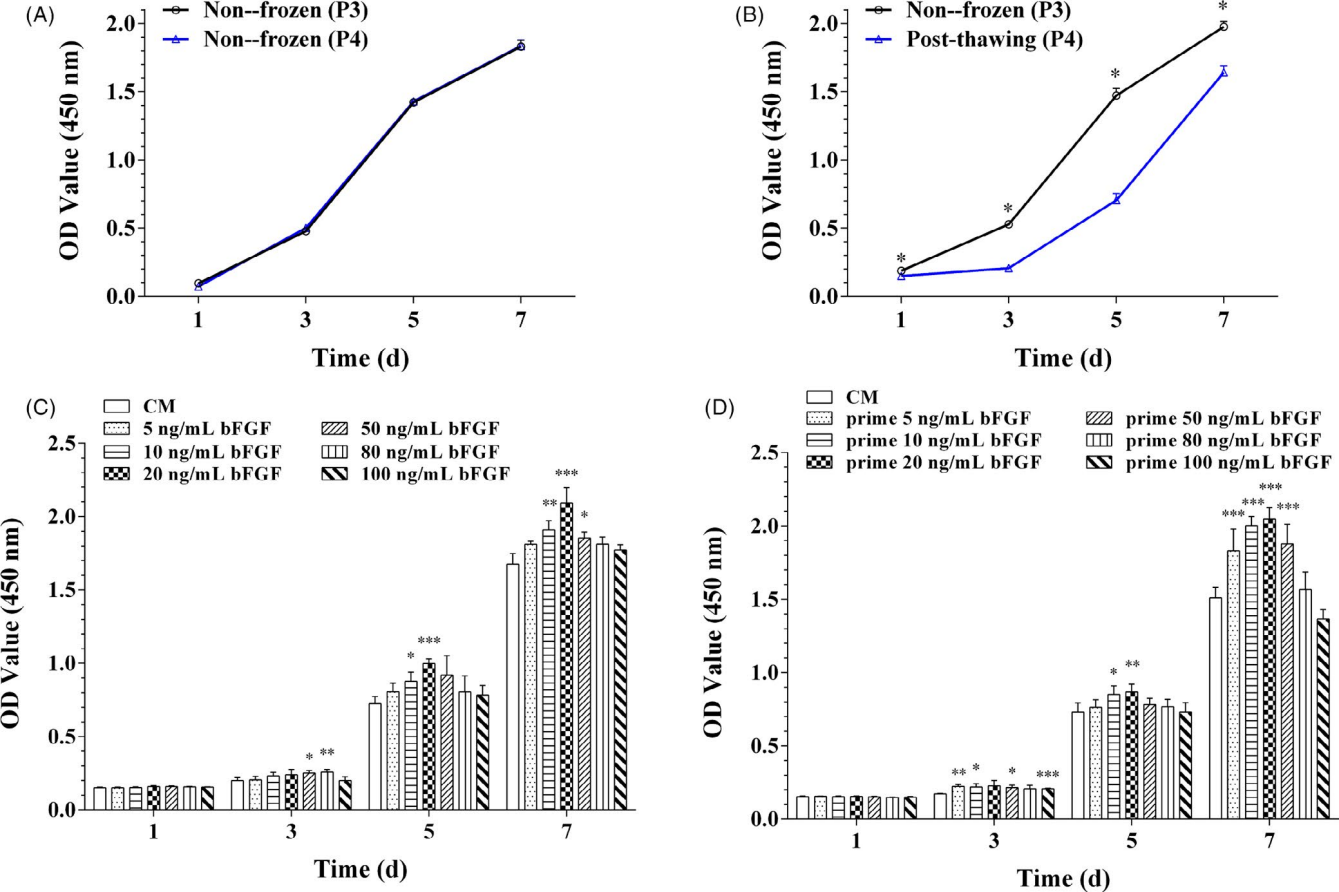
The cellular growth curve before and after cryopreservation, and cell proliferation of post‐thawed DPSCs and their succeeding passage. (A) The cell proliferation of non‐frozen DPSCs between the passage 3 (P3) and the passage 4 (P4) from day 1 to 7. No statistical differences were observed between the two passages at all timepoints. Data were represented as mean ± SD (n = 3) (B) The cellular viability of post‐thawed DPSCs (P4) was statistically lower compared to the before‐frozen cells (P3) from day 1 to 7. All data were represented as mean ± SD (n = 3), ^*^ *P* < .05. (C) Cell proliferation of post‐thawed DPSCs (P4) in the 20 ng/mL bFGF group was higher than other groups on day 5 and 7. (D) After supplemented with bFGF for 5 d, DPSCs (P4) were passaged to P5 and cultured with bFGF‐free medium. The succeeding passage (P5) also proliferated fastest in the 20 ng/mL bFGF group on day 5 and 7. All data were represented as mean ± SD (n = 3), ^*^ *P* < .05, ^**^ *P* < .01 and ^***^ *P* < .01 vs the control (CM)

The CCK‐8 results displayed the growth curve of DPSCs before freezing and after cryopreservation. As shown in Figure 1A, the cell proliferation of non‐frozen DPSCs had no significantly different between the passage 3 (P3) and passage 4 (P4) when tested from day 1 to 7. However, the results illustrated that the cellular viability of post‐thawed DPSCs (P4) was statistically lower compared to the before‐frozen cells (P3) from day 1 to 7 (*P* < .05, Figure 1B).

### 3.3 bFGF promoted the cell proliferation of post‐thawed DPSCs and its succeeding passage

The CCK‐8 results indicated that the proliferation of post‐thawed DPSCs (P4) in the 20 ng/mL bFGF group was significantly higher than other six groups from day 5 to 7 (*P* < .05, Figure 1C). After 5‐day bFGF primed culture, DPSCs cells (P5) were cultivated in bFGF‐free complete medium. Data showed that the effects of bFGF on cellular proliferation extended to their succeeding passages, cells in the 20 ng/mL bFGF group proliferated faster than those in other groups from day 5 to 7 (*P* < .05, Figure 1D). Hence, 20 ng/mL bFGF was regarded as an optimal concentration and chosen to perform following studies.

### 3.4 bFGF impacted on apoptosis and TRPC1 channel of post‐thawed DPSCs

**Figure 2:**
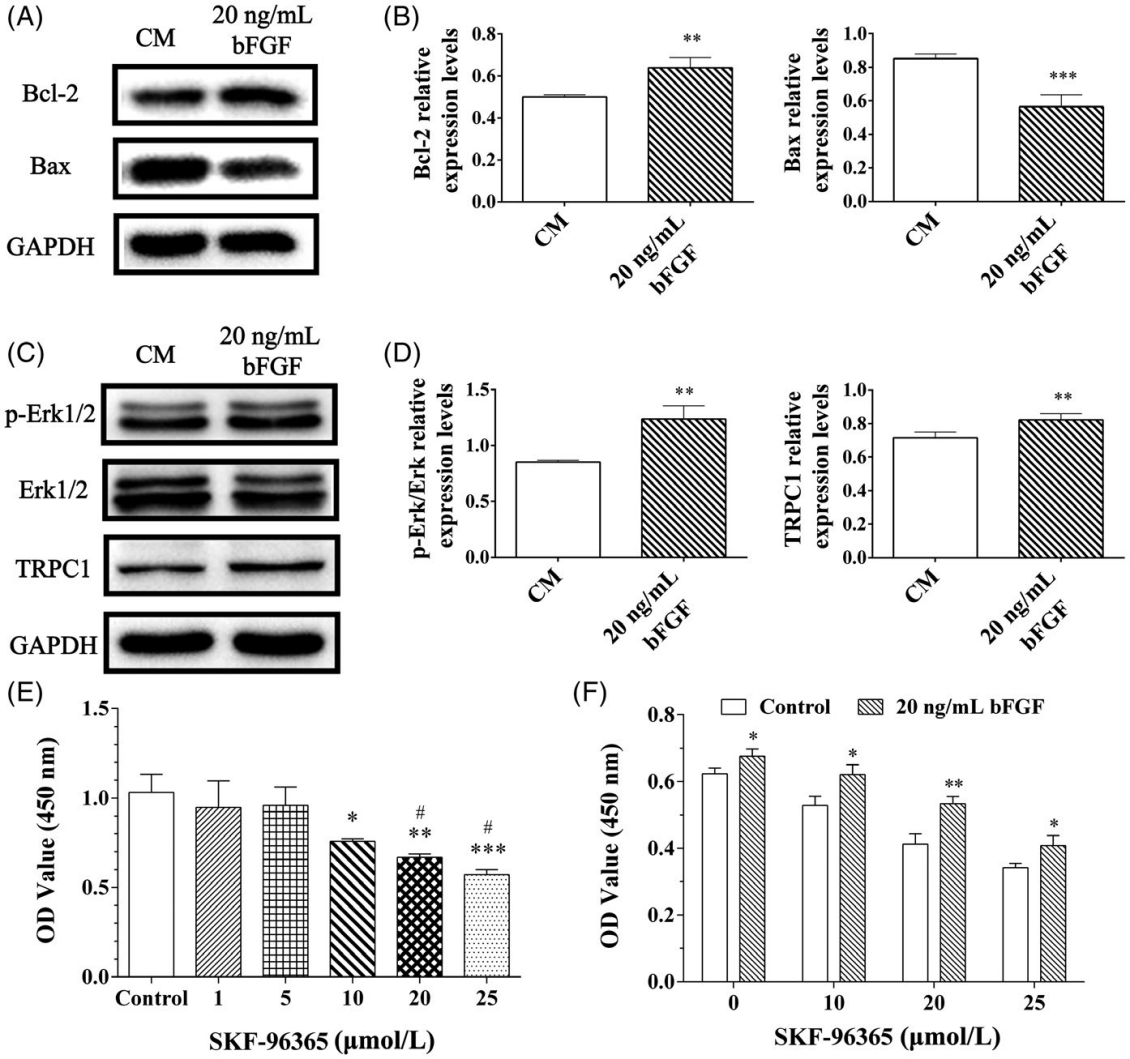
bFGF impacted on apoptosis of post‐thawed DPSCs and TRPC1 channel, and bFGF rescued cellular proliferation after TRPC1 inhibition. (A, C) Western blot analysis of Bcl‐2, Bax, p‐Erk1/2, Erk1/2 and TRPC1 expression in DPSCs with/ without bFGF treatment. (B, D) Quantification of these proteins in DPSCs with bFGF treatment indicated that down‐expression of Bax, up‐expression of Bcl‐2, p‐Erk1/2, Erk1/2 and TRPC1. All data were represented as mean ± SD (n = 3), ^**^ *P* < .01, ^***^ *P* < .001 vs the control (CM). (E) TRPC1 inhibitor, SKF‐96365, effectively suppressed the proliferation of DPSCs (P4) at 10‐25 µmol/L. (F) DPSCs (P4) were pre‐treated with SKF‐96365 for 24 h and then cultured with/ without 20 ng/mL bFGF for another 24 h. Additional bFGF significantly increased the cell proliferation in a short‐term culture. ^*^ *P* < .05, ^**^ *P* < .01 and ^***^ *P* < .001 vs the control group; ^#^ *P* < .05 vs the 1 μmol/L group. All data were represented as mean ± SD (n = 5)

After exposed to 20 ng/mL bFGF for 3 d, in comparison with the control group (CM, bFGF‐free), statistically high expression of Bcl‐2 (anti‐apoptotic factor) and low expression of Bax (pro‐apoptotic factor) were observed in the bFGF group (*P* < .05, Figure 2A,B). The cell proliferation‐related proteins, p‐Erk/Erk and TRPC1, were both highly expressed in bFGF group (*P* < .05, Figure 2C,D).

To explore whether TRPC1 channel plays a major role in the bFGF‐induced proliferation of post‐thawed DPSCs (P4), SKF‐96365, the TRPC1 inhibitor, was added to the medium and analysed by CCK‐8. It is indicated that 10‐25 μmol/L SKF‐96365 could effectively suppress the proliferation of DPSCs in a dose‐dependent manner (Figure 2E). And addition of 20 mg/mL bFGF could significantly rescue the proliferation of TRPC1 inhibited DPSCs (*P* < .05, Figure 2F).

### 3.5 Stemness of the succeeding passage from bFGF pre‐treated DPSCs

**Figure 3:**
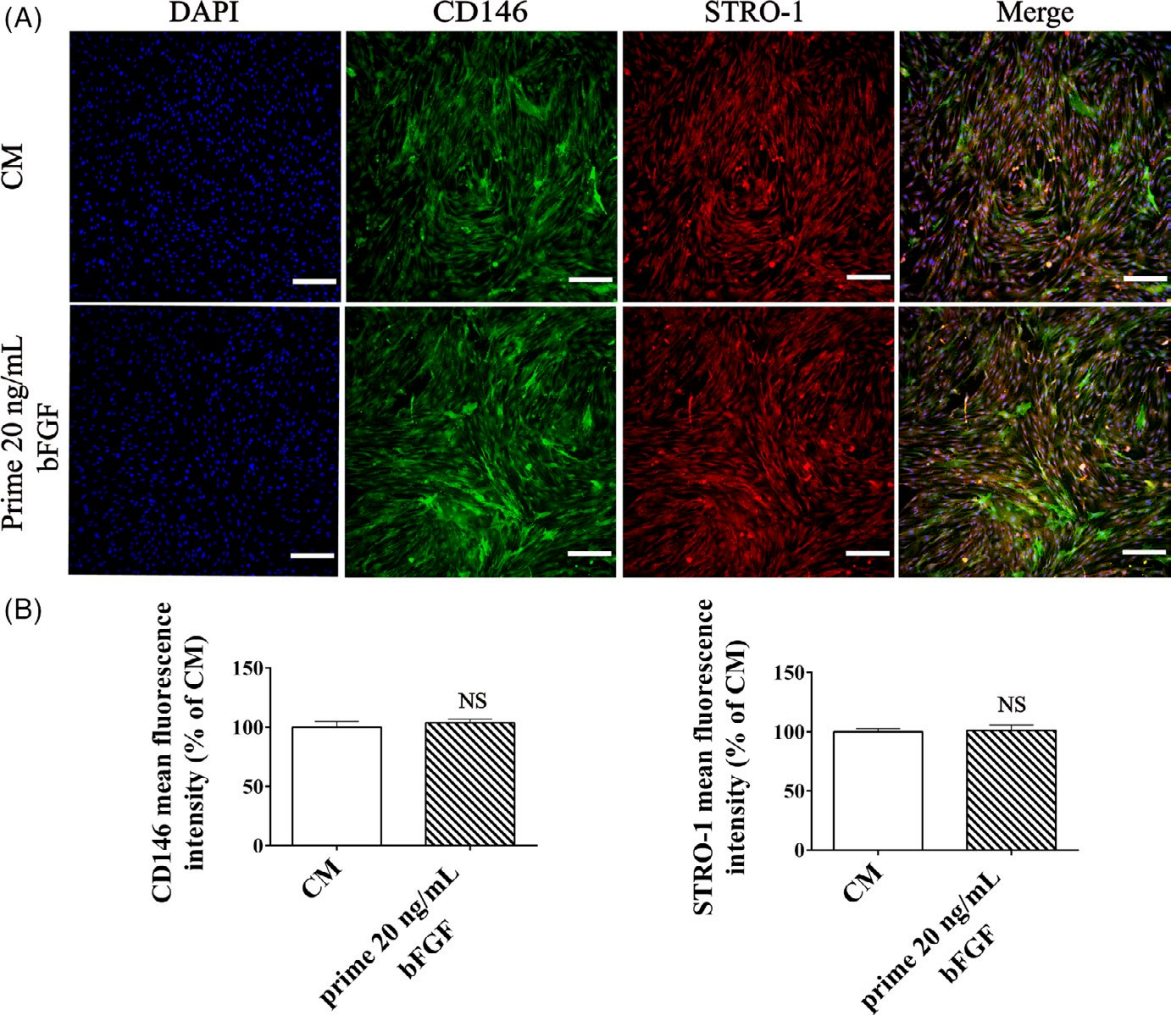
Immunofluorescence labelled MSCs markers of the succeeding passage from bFGF pre‐treated DPSCs. (A) Post‐thawed DPSCs (P4) were firstly cultured with bFGF (20 ng/mL) for 5 d and passaged to P5, then stained with CD146 (green), STRO‐1 (red) and nucleus (blue). DPSCs (P5) in CM group were cultured in bFGF‐free medium. *Scale bar*: 200 μm. (B) Semi‐quantification of the fluorescence intensity of CD146 and STRO‐1. There is no statistical difference (NS) in the expression of these surface markers between bFGF pre‐treated and control DPSCs (P5). All data were represented as mean ± SD (n = 3)

**Figure 4:**
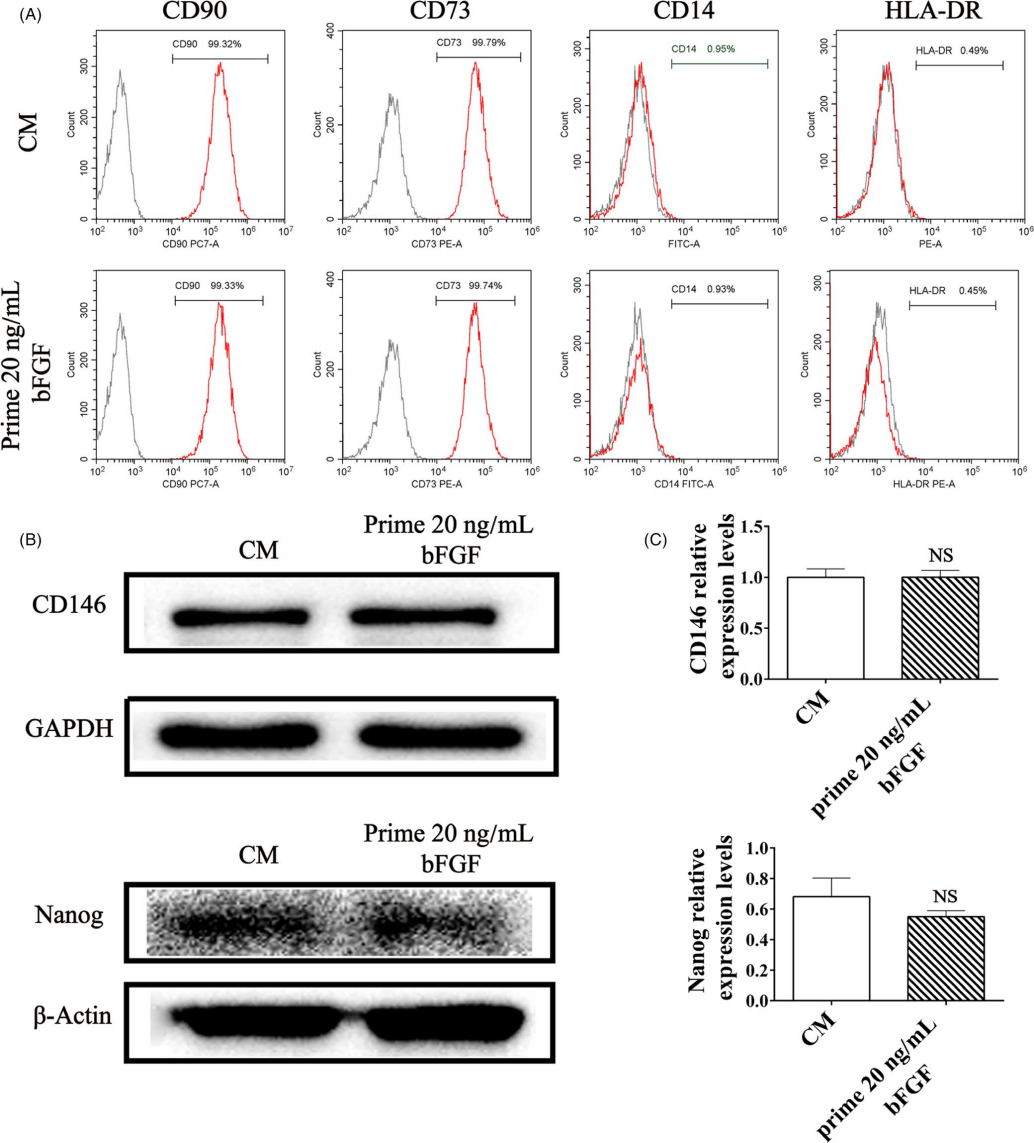
Stemness of the succeeding passage from bFGF pre‐treated DPSCs. (A) Post‐thawed DPSCs (P4) were firstly cultured with bFGF (20 ng/mL) for 7 d and passaged to P5, then assessed the MSCs markers (CD73 and CD90) and haematopoietic stem cells (CD14 and HLA‐DR). The expression of these markers between bFGF pre‐treated and control DPSCs (P5) was similar. (B, C) Quantification of the proteins of CD146 and Nanog in bFGF pre‐treated and control DPSCs (P5) indicated no statistical difference (NS). All data were represented as mean ± SD (n = 3)

Post‐thawed DPSCs (P4) were supplemented with 20 ng/mL bFGF for 7 days and passaged to P5 with bFGF‐free culture. Cells in the control group were treated with complete medium (CM). Surface markers of MSCs, CD146 and STRO‐1, both were positive staining (Figure 3A), and the fluorescence intensity in bFGF pre‐treated group did not differ from control group (*P* > .05, Figure 3B). Flow cytometry identified equivalent expression level of MSC markers in the bFGF pre‐treated DPSCs and the control group (Figure 4A). Western blotting showed that the expression levels of CD146 and Nanog in bFGF pre‐treated DPSCs did not statistically differ from the control group (*P* > .05, Figure 4B,C). Thus, supplement with bFGF in culture of DPSCs did not compromise the stemness of succeeding passage.

### 3.6 Pluripotency of the succeeding passage from bFGF pre‐treated DPSCs

**Figure 5:**
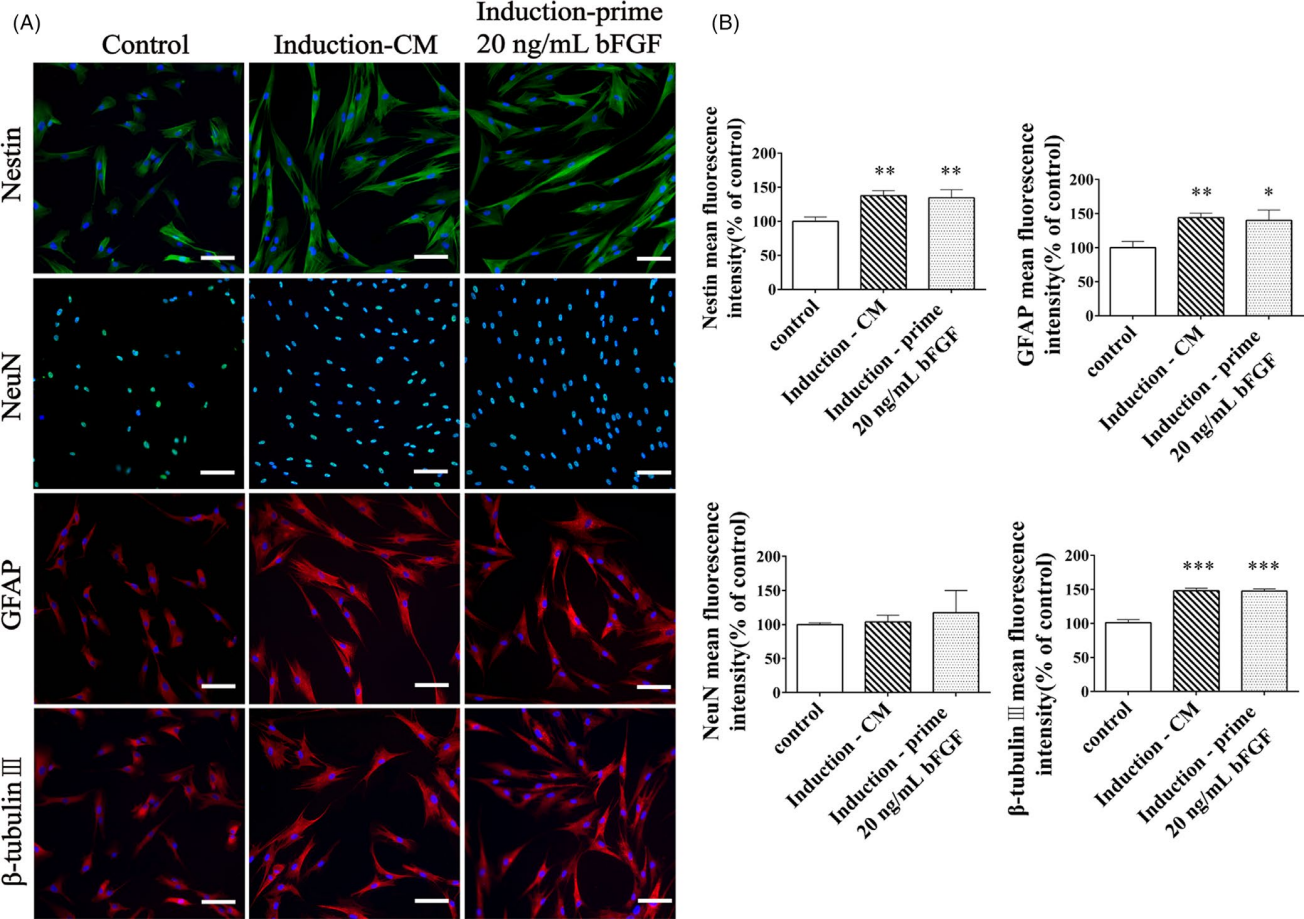
Neurogenic differentiation of the succeeding passage from bFGF pre‐treated DPSCs. In the Control group, DPSCs (P5) were cultured in bFGF‐free medium and not induced. In the Induction‐CM group, cells (P5) were cultured without bFGF and induced. In the Induction‐prime 20 ng/mL bFGF group, cells (P5) were passaged from bFGF pre‐treated DPSCs and induced. (A) Immunofluorescent staining showed neural markers of DPSCs (P5), including Nestin (green), NeuN (green), GFAP (red) and β‐tubulin III (red). Cell nuclei were stained with DAPI (blue). *Scale bar*: 100 μm. (B) Semi‐quantification of the fluorescent intensity of these neural markers. The induction enhanced these expressions, and the expression level of bFGF pre‐treated DPSCs (P5) was similar to the cells in bFGF‐free culture. All data were represented as mean ± SD (n = 3), ^*^ *P* < .05, ^**^ *P* < .01 and ^***^ *P* < .001 vs the Control group

**Figure 6:**
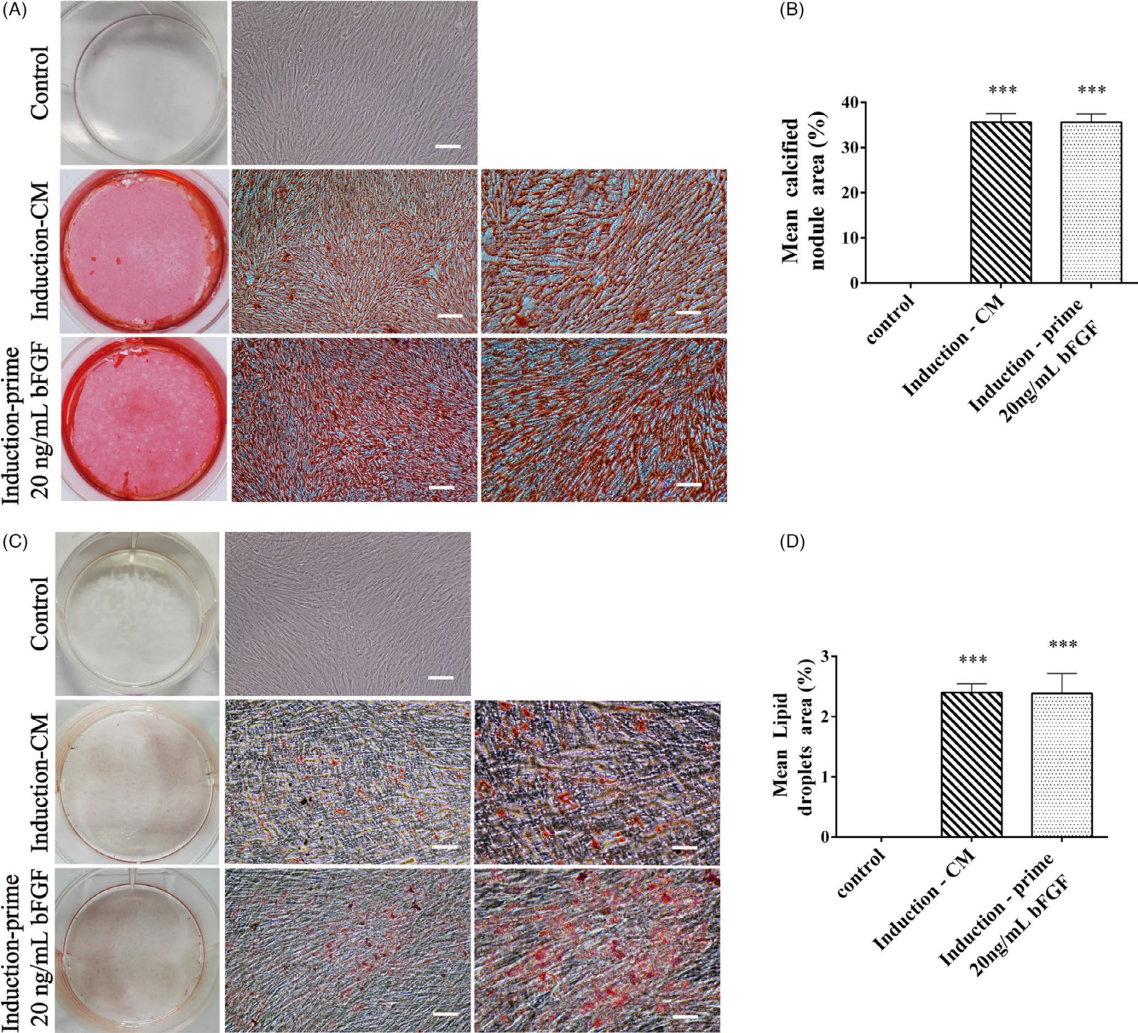
Osteogenic and adipogenic differentiation of the succeeding passage from bFGF pre‐treated DPSCs. In the Control group, DPSCs (P5) were cultured in bFGF‐free medium and not induced. In the Induction‐CM group, cells (P5) were cultured without bFGF and induced. In the Induction‐prime 20 ng/mL bFGF group, cells (P5) were passaged from bFGF pre‐treated DPSCs and induced. In the Control, cells did not display newly regenerated structure (top panel in A and C). At low and high magnification of induction groups (middle and right column in A and C), mineralized nodules were stained by Alizarin Red and lipid droplets were stained by Oil Red O *Scale bar*: 200 μm and 100 μm. (B, D) Semi‐quantification of the newly regenerated structure. Induction groups sustained similar potential to differentiate into osteoblast or adipocyte. All data were represented as mean ± SD (n = 3), ^***^ *P* < .001 vs the Control group

DPSCs (P5) were cultured in the same methods as the stemness study and their pluripotency was analysed. DPSCs were inherently expressed of Nestin, NeuN, GFAP and β‐tubulin III in bFGF‐free control, un‐induced group (Figure 5A, left column). After neural induction, both bFGF and CM pre‐treated DPSCs showed up‐regulation of these markers (except NeuN) compared with the un‐induced group (*P* < .05), and both induction groups illustrated the similar expression of neural markers (*P* > .05, Figure 5B). To assess the osteogenic and adipogenic differentiations, Alizarin Red and Oil Red O were used to stain mineralized nodules and lipid droplets, respectively (Figure 6A,C). After lineage induction, both bFGF and CM pre‐treated DPSCs displayed newly regenerated structures compared with the bFGF‐free control, un‐induced group (*P* < .05), and both induction groups displayed the comparable amounts of nodules and lipids (*P* > .05, Figure 6B,D). Hence, 20 ng/mL bFGF pre‐treatment did not affect the pluripotency of the succeeding passage from post‐thawed DPSCs.

## 4 DISCUSSION

DPSCs obtained by enzymatic digestion methods form heterogeneous cell population. Compared to the negative CD146 (CD146^−^) and unpurified cells, CD146^+^ DPSCs showed higher osteogenic, adipogenic and chondrogenic potential.^35^, ^36^ Meanwhile, CD146^+^ DPSCs had high proliferation rate, and the doubling time of DPSCs is calculated as 13‐31 hours, which was consistent with the previously described.^37^ Our results indicated that CD146^+^ DPSCs could maintain stable and high proliferation activity for a long time (Figure S3). Thus, CD146^+^ DPSCs were the promising stem cell sources for tissue engineering and regenerative medicine. And access to homogeneous CD146^+^ DPSCs population makes it possible to obtain consistent results in both basic and clinical studies. Moreover, application of stem cells usually relies on prolonged storage, for example cryopreservation, so it is critical for post‐thawed DPSCs to maintain excellent proliferative and regenerative capacity as they often suffer from cell cycle cessation and proliferation deceleration (Figure 1A,B).

bFGF participates in proliferation and self‐renewal of stem cells.^38^, ^39^ Previous study demonstrated that 5 ng/mL bFGF effectively enhanced the proliferation of bone marrow mesenchymal stem cells.^31^ And we discovered that 20 ng/mL bFGF significantly boosted up the viability of post‐thawed DPSCs, and this advantageous effect emerged at day 5 (Figure 1C). It is implied that the optimal concentration of bFGF to stimulate proliferation may differ from cell type and status. When compared to the bFGF‐free group, we found this proliferative superiority could be sustained in the succeeding passages of bFGF pre‐treated DPSCs (20 ng/mL, 5 days) (Figure 1D).

The anti‐apoptosis effect of bFGF partially involves the ERK pathway.^40^ Also, bFGF enhances cellular proliferation and migration via the PDGFRb/p‐Erk pathway,^41^ and promotes self‐renewal by the Akt and Erk1/2 pathways.^42^ Our work showed that addition of bFGF could regulate apoptosis and viability of post‐thawed DPSCs at early stage, evidenced by up‐expression of Bcl‐2 and down‐expression of Bax (Figure 2A,B), and promoted the volume of p‐Erk/Erk protein and TRPC1 channel (Figure 2C,D) at day 3. Meanwhile, the advantageous reaction of 20 ng/mL bFGF on cell growth emerged at day 5. It is illustrated that the anti‐apoptosis and survival‐supporting effect of bFGF on the post‐thawed DPSCs might display earlier than the exhibition of proliferation‐boosting responses.

To explore whether bFGF influenced cell proliferation via TRPC1 channel, SKF 96365 was used to block the TRPC1.^43^ Our data discovered that SKF‐96365 could effectively suppress the proliferation of DPSCs and this inhibition could be reversed by bFGF (Figure 2E,F). It is reported that Erk1/2 activation associates with TRPC1 overexpression.^44^ Meanwhile, bFGF signals could transduce through the ERK pathway. Therefore, the mechanism underlying the role of bFGF on DPSCs proliferation possibly associated with positive regulation of TRPC1 channel via triggering the ERK pathway and altering the expression of apoptosis‐related proteins, but the clear relationship among the ERK pathway, TRPC1 channel and apoptosis remained unknown and needed further study.

Then, we investigated the regenerative properties of the succeeding passage of bFGF pre‐treated DPSCs. Previous study indicated that post‐thawed MSCs might suffer from differentiation compromise,^45^ yet we found that post‐thawed DPSCs remained MSCs markers (99.79% CD73 and 99.32% CD90) and haematopoietic stem cell markers (0.95% CD14 and 0.49% HLA‐DR) (Figure 4A), similar to the unfrozen cells (Figure S2E). It is demonstrated that post‐thawed DPSCs kept stemness characteristics.^46^ Moreover, bFGF would increase the fraction of Stro‐1^+^/CD146^+^ progenitor cells,^47^ and our data displayed that the succeeding passage of bFGF pre‐treated DPSCs could sustain MSCs early markers^48^ such as CD146 and STRO‐1 (Figure 3). As far as in situ applications are concerned, controlled release nano‐delivery systems could be used to maintain the bFGF concentration.^49^, ^50^, ^51^

In multi‐lineage differentiation studies, notably, to eliminate the disturbance from growth factors, we adopted chemical‐induction protocols to assess neurogenic differentiation ability of the succeeding passage from bFGF pre‐treated DPSCs.^34^ We found that supplement with bFGF did not compromise the pluripotency of DPSCs to differentiate into neurons, osteoblasts and adipocytes (Figures 5 and 6).

bFGF is an important factor for cell activities which plays pivotal roles in cell growth, proliferation and differentiation. Some studies have shown that the application of bFGF could facilitate the cells attachment, growth and proliferation, and temporarily inhibit the multi‐differentiation of stem cells at the early using phase.^52^, ^53^ Meanwhile, short‐term bFGF treatment could enhance the stemness of dental stem cells.^17^, ^18^ Moreover, bFGF‐activated FGFRs could be used to transduce the signals to maintain the pluripotency of stem cells.^19^, ^20^ However, little has been known on the succeeding passage from bFGF pre‐treated DPSCs after cryopreservation. Our data demonstrated that temporary application of bFGF at the early stage of post‐thawing could rescue cellular viability without compromising their stemness and pluripotency.

In this work, we proved the safety and feasibility of supplement with bFGF in culture medium to amplify post‐thawed DPSCs rapidly. In 3‐month storage, the innate stemness of DPSCs remained; yet the proliferation was restrained due to the activation of apoptosis. Adding bFGF (20 ng/mL) immediately in revived culture could significantly enhance cell proliferation via activating the ERK pathway, up‐regulating TRPC1 channel and preventing apoptosis. And we also detected the regenerative property of their succeeding passage which showed satisfying growth rate, stemness and pluripotency. It is demonstrated that short‐term application of bFGF in the early stage post‐thawing could rescue cellular viability without shifting their pluripotency. Remarkably, such proliferative superiority could prolong to the succeeding passage, so the usage time of bFGF for post‐thawed cell culture should be addressed. Nowadays, DPSCs‐based therapy was proceeding into an advanced stage, ranged from in vitro to in vivo studies. To satisfy the growing needs in scientific and clinical applications, DPSCs cryo‐storage facilities and services, a.k.a. dental stem cell banking, have established in many countries. Clearly, our study provided cues and clues for cell culture strategy to rapidly amplify post‐thawed DPSCs with robust regenerative potency, which brightens the future of DPSCs banking and tissue engineering.

## CONFLICT OF INTEREST

The authors declare that they have no competing interests regarding the publication of this paper.

## AUTHOR CONTRIBUTIONS

Lihua Luo and Yanni Zhang performed the majority of experiments and analysed the data; Xiaoyan Wang and Fengting Hu collected the data and performed the statistical analysis; Zhenjie Xing, Hongyu Chen and Abdullkhaleg Ali Albashari performed the multi‐differentiation of DPSCs; Qingsong Ye and Yan He designed and coordinated the research; Lihua Luo and Jian Xiao wrote the paper.

## Supporting information

Fig S1

Fig S2

Fig S3

Supplementary Material

## Data Availability

The datasets for this study are available on request to the corresponding authors.
