# Supplementary figures and images for: Effects and mechanisms of basic fibroblast growth factor on the proliferation and regenerative profiles of cryopreserved dental pulp stem cells
Source: Cell Prolif. 2020 Dec 17;54(2):e12969. doi: 10.1111/cpr.12969 (PMC7848956; doi:10.1111/cpr.12969)

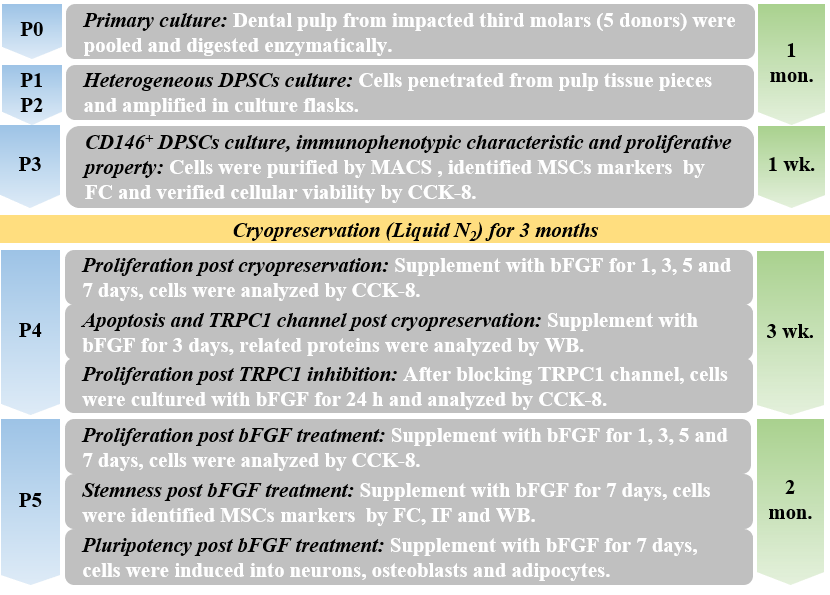

Supplement: Supplementary file 1 — Fig S1 [file CPR-54-e12969-s001.tif]

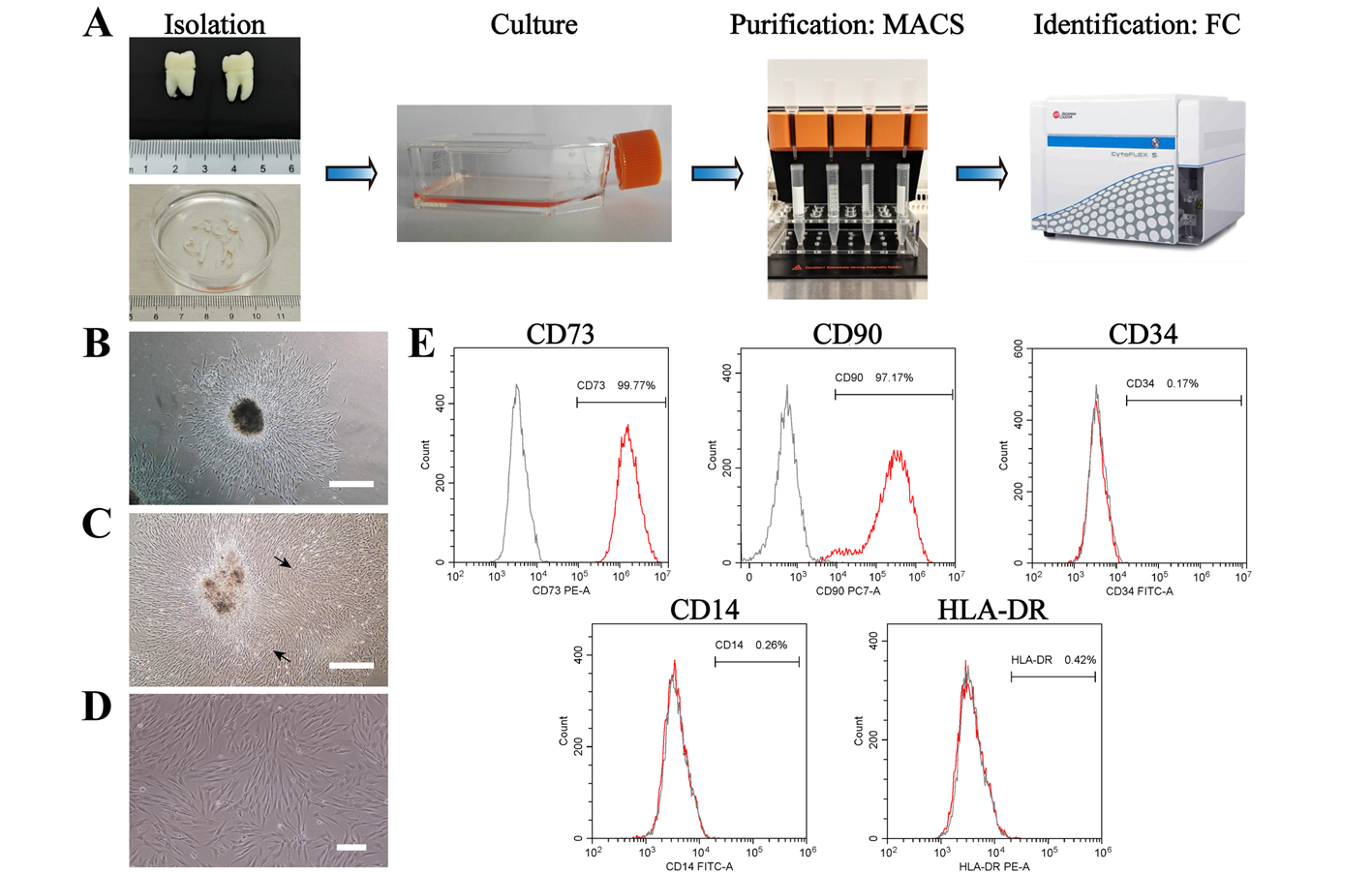

Supplement: Supplementary file 2 — Fig S2 [file CPR-54-e12969-s002.tif]
