# Supplementary material for: Effects and mechanisms of basic fibroblast growth factor on the proliferation and regenerative profiles of cryopreserved dental pulp stem cells
Source: Cell Prolif. 2020 Dec 17;54(2):e12969. doi: 10.1111/cpr.12969 (PMC7848956; doi:10.1111/cpr.12969)
Supplement: Supplementary file 4 — Supplementary Material [file CPR-54-e12969-s004.docx]

Supplementary data:

Effects and mechanisms of basic fibroblast growth factor on the proliferation and regenerative profiles of cryopreserved dental pulp stem cells

*Running title:*

**Effects of bFGF on cryopreserved DPSCs**

Lihua Luo^1†^, Yanni Zhang^1†^, Hongyu Chen^2†^, Fengting Hu^1^, Xiaoyan Wang^1^, Zhenjie Xing^1^, Abdullkhaleg Ali Albashari^1^, Jian Xiao^3^, Yan He^4*^, Qingsong Ye^1,5*^

^1^School and Hospital of Stomatology, Wenzhou Medical University, Wenzhou, Zhejiang, China

^2^Department of Stomatology, Ningbo Women and Children Hospital, Ningbo, China

^3^School of Pharmaceutical Sciences, Wenzhou Medical University, Wenzhou, China

^4^Skeletal Biology Research Center, Massachusetts General Hospital, Harvard University, Boston, USA

^5^Center of Regenerative Medicine, Renmin Hospital of Wuhan University, Wuhan, China

***Correspondence:** Yan He, helen-1101@hotmail.com; Qingsong Ye, qingsongye@hotmail.com;

† These authors have contributed equally to this work

Keywords: Dental pulp stem cells, Basic fibroblast growth factor, Transient receptor potential canonical 1 channel, Extracellular signal-regulated kinase pathway, Cryopreservation, Cell culture technique

**FIGURE. S1** Workflow and timeline of the whole experiment.

**FIGURE. S2** Isolation, culture, purification and identification of DPSCs. (A) After primary culture (P0), heterogeneous DPSCs (P2) were separated by magnetic-activated cell sorting (MACS) to obtain CD146^+^ DPSCs (P3), then characterized via flow cytometry (FC). (B) Colony-forming units reached low density on day 7. (C) Representative high density of colonies (black arrows) on day 10. (D) Typical fibroblast-like morphology of DPSCs (P1). (E) Immunophenotype of CD146^+^ DPSCs. Markers were demonstrated as red and respective isotypes were in gray. *Scale bars*: A and B, 500 μm; C, 200 μm.

**FIGURE. S3** The cell proliferation at day 3 of non-frozen DPSCs from the passage 3 (P3) to the passage 7 (P7). No statistical difference were found among these passages. All data were represented as mean ± SD (n=3).
